# Prevalence and determinants of medications non-adherence among patients with uncontrolled hypertension in primary care setting in Sarawak, Malaysia: A cross-sectional study

**DOI:** 10.51866/oa.182

**Published:** 2022-10-26

**Authors:** Hui Zhu Thew, Siew Mooi Ching, Hooi Min Lim, Mike Hitler Mos, Lorna Chin Kin Tze, Kui Feng Low, Nurdarlina Shaari, Jody Yii Sze Lin, Kai Wei Lee, Vasudevan Ramachandran

**Affiliations:** 1 MD (UNIMAS), MMed (UM), Department of Family Medicine, Faculty of Medicine and Health Sciences, Universiti Putra Malaysia, Serdang, Malaysia.; 2 Malaysian Research Institute on Ageing (MyAgeing), Universiti Putra, Malaysia, Serdang, Malaysia.; 3 Department of Medical Sciences, School of Healthcare and Medical Sciences, Sunway University, 5 Jalan University, Bandar Sunway, Selangor Darul Ehsan, Malaysia.; 4 MD (NNSMA), MMed (UPM), Department of Family Medicine, Faculty Medicine and Health Sciences, Serdang, Malaysia.; 5 MBBS (UM), MMed (UM), Department of Primary Care Medicine, Faculty of Medicine, University of Malaya, Kuala Lumpur, Malaysia.; 6 MBBS (UM), Klinik Kesihatan Batu Kawa, Jalan Ensing Timur, Off Jalan, Stapok Utama,Kuching, Sarawak, Malaysia.; 7 MD (UPM), MAFP, FRACGP, Klinik Kesihatan Miri, Jalan Merbau, Miri, Sarawak, Malaysia.; 8 MD, Klinik Kesihatan Jalan Masjid, Jalan Masjid, Kuching, Sarawak, Malaysia.; 9 MBBS(UM), MAFP, FRACGP, Klinik Kesihatan Telaga Air, Jalan Matang, Kuching, Sarawak, Malaysia.; 10 MD (UNIMAS), MAFP, FRACGP, Klinik Kesihatan Tudan, Jalan Permyjaya, Miri, Sarawak, Malaysia.; 11 Ph.D. (UPM), Department of Medical Microbiology, Faculty of Medicine and Health Sciences, Universiti Putra, Malaysia, Serdang, Malaysia.; 12 Ph.D. (UPM), Faculty of Health Sciences, University College MAIWP, International College, Taman Batu, Muda, Batu Caves, Kuala Lumpur, Malaysia.

**Keywords:** Patient compliance, Hypertension, Primary health care, Malaysia

## Abstract

**Introduction:**

Non-adherence to antihypertensive medications is a leading cause of uncontrolled hypertension and its complications. However, data on the factors associated with non-adherence to antihypertensive medications in the communities of Sarawak, Malaysia, are limited. This study aimed to examine the prevalence and determinants of medication non-adherence among patients with uncontrolled hypertension.

**Method:**

A cross-sectional study was conducted using the systematic sampling method in four government primary healthcare clinics in Sarawak. A self-administered questionnaire was used to obtain socio-demographic data and evaluate non-adherence. Blood pressure was measured, and relevant clinical variables were collected from medical records. Multivariate logistic regression was used to determine the determinants of medication non-adherence.

**Results:**

A total of 488 patients with uncontrolled hypertension were enrolled in this study. The prevalence of medication non-adherence was 39.3%. There were four predictors of medication non-adherence among the patients with uncontrolled hypertension: tertiary educational level (odds ratio [OR]=4.21, 95% confidence interval [CI] = 1.67–10.61, P=0.010), complementary alternative medication (0R=2.03, 95% CI=1.12–3.69, P=0.020), non-usage of calcium channel blockers (0R=1.57, 95% CI=1.02–2.41, P=0.039) and 1 mmHg increase in the systolic blood pressure (0R=1.03, 95% CI=1.00–1.05, P=0.006).

**Conclusion:**

Because of the high prevalence of medication non-adherence among patients with uncontrolled hypertension, primary care physicians should be more vigilant in identifying those at risk of being non-adherent. Early intervention should be conducted to address non-adherence for blood pressure control.

## Introduction

Hypertension is the most common health condition seen in primary healthcare settings, which can lead to myocardial infarction, stroke, renal failure and death if not detected early and treated appropriately.^1^ It is an increasing global health problem in developed and developing countries, including Malaysia. Globally, 30.8% of adults have hypertension, and the estimated prevalence of hypertension among men and women is 32.1% and 29.5%, respectively.^2^ In Malaysia, the prevalence of hypertension plateaued at 30%,^3,4^ which is higher than that in other countries, including China and Singapore.^5^ The prevalence trend in Malaysia is more similar to that in developed countries than in developing countries.^6^ Hypertension is the greatest contributor to the overall mortality in Malaysia in terms of disability-adjusted life-years, with mortality rates of 19.4% in men and 22.8% in women.^7^ Hypertension, especially uncontrolled and untreated hypertension, is associated with an increased risk of total and cardiovascular mortalities among the general population with hypertension.^8^ The main goal of hypertension management is to prevent end-organ damage and delay the complication progression. This could be achieved mainly through blood pressure control and cardiovascular risk stratification.^9^ The complications indirectly increase healthcare costs to both the patient and the community.^10,11^ The causes of uncontrolled hypertension can be classified into three main factors: patient-, physician- and healthcare system-related causes.^12-14^ Among all these factors, poor medication adherence is the top contributor to uncontrolled blood pressure.^12^ The causes of non-adherence can also be classified into patient-, physician- and healthcare system-related factors.^13,15^ Patient forgetfulness, lack of information related to hypertension and its complications, emotional factors, fear of addiction to medications or side effects of antihypertensive medications, complexity of treatment, missed appointments and lack of belief in the benefit of medications are among the patient-related factors of non-adherence.^15-17^

Changes in lifestyle and a demographic shift in Sarawak, Malaysia, have contributed to the increased prevalence of hypertension. A national survey conducted in 2004 in Malaysia showed that the prevalence of hypertension in respondents aged 30 years and above was 40.5% (39.3%-41.8%).^3,18^ Malays and the indigenous people from the state of Sabah had the highest prevalence of hypertension at 41.3%, followed by the indigenous people of Sarawak (40.4%), Chinese (40.0%) and Indians (37.7%).^18^ However, only one study was conducted among elderly populations with hypertension in Miri, Sarawak, to evaluate medication adherence.^15^ This shows that studies examining medication non-adherence among patients with uncontrolled blood pressure in a community such as Sarawak, where the main ethnicity is an indigenous group in Malaysia, are limited.^15^ Thus, the present study aimed to examine the prevalence and determinants of medication non-adherence among patients with uncontrolled blood pressure.

## Methods

### Setting

A cross-sectional study was conducted from August to September 2019 among patients who attended four government primary care clinics in Sarawak (Klinik Kesihatan Oya, Klinik Kesihatan Miri, Klinik Kesihatan Jalan Masjid and Klinik Kesihatan Batu Kawa). All these four clinics are located in the city centre, and each clinic accommodates an estimated 600 to 800 patients per day.

### Inclusion criteria

The following patients were eligible for inclusion to the study: patients with uncontrolled hypertension diagnosed in accordance with the Malaysian Clinical Practice Guidelines for Hypertension (blood pressure of ≥140/90 mmHg for patients with hypertension, ≥140/80 mmHg for patients with diabetes, ≥130/80 mmHg for patients with ischaemic heart disease/cerebrovascular disease and renal impairment and ≥150/90 mmHg for patients aged >80 years)^9^; those aged 30 years and above; those taking one or more antihypertensive medications; and those with a minimum follow-up period of 6 months at Klinik Kesihatan Oya, Klinik Kesihatan Miri, Klinik Kesihatan Jalan Masjid and Klinik Kesihatan Batu Kawa.

### Exclusion criteria

Patients who refused to provide consent, pregnant mothers, acutely ill or unstable patients, and those who were diagnosed with secondary hypertension were excluded.

### Sample size calculation

The sample size was calculated using Epi Info version 7.0 based on the local study by Ramli et al. in which the prevalence of medication non-adherence was 46.6%.^16^ The calculated sample size was 381, with 95% power, 95% confidence interval (CI) and 5% α value. The final sample size was 457 after considering a 20% dropout rate.

### Sampling method

Systematic random sampling was used for recruitment. Twelve patients with uncontrolled blood pressure were estimated to visit each clinic in a day. Hence, 480 patients with uncontrolled hypertension were estimated to visit each clinic within 8 weeks. With the required sample size of 114 (457/4) in each clinic, the sampling interval of four was used, with the starting number being picked randomly from number one to four using the lottery method from the registration counter. Patients were recruited directly inside the hypertension room after registration with a designated hypertension clinic. Meanwhile, nurses at the registration counter sent the patients to the investigation room on the day of data collection at the clinic.

### Pilot study

The study questionnaire was pre-tested among 50 patients to check its comprehensibility and test the data collection process. Minor revisions, especially the questionnaire’s alignment, were made following the pilot study. The Cronbach’s α was not evaluated after the study, as the questionnaire has been previously validated locally (Cronbach’s α of 0.782).

### Data collection instrument

A self-administered questionnaire was used for data collection. The questionnaire has four parts. Part one contains questions regarding demographic characteristics. Part two contains items on hypertension characteristics and was filled out by the patients and researchers. For blood pressure measurement, the patients were instructed to sit upright without crossing their legs and rest for 5 minutes. They were instructed refrain from exercising, drinking caffeinated beverages, eating or smoking 30 minutes before the measurement. An appropriate-sized cuff that encircled at least 80% of the arm, with the bladder covering at least 40% of the arm circumference, placed 2 cm above the brachial artery and aligned with the index marker, was used. A sphygmomanometer was placed at the same level as the patients’ heart, and the systolic and diastolic measurements were recorded.

Part three contains the modified Medication Adherence Scale, while part four contains questions regarding healthcare systems. The modified Medication Adherence Scale was developed by Ramli et al.^16^ based on two different adherence questionnaires: the Hill— Bone Adherence to Blood Pressure Therapy Scale and Morisky Medication Adherence Scale. The modified Medication Adherence Scale has been validated locally with Cronbach’s α of 0.782 and had good internal consistency and reliability.^16^ There are seven items in this questionnaire, and each question has a four-point Likert-type response format. Each response carried a score: none of the time=4, some of the time=3, most of the time=2 and all the time=1. The total score for each patient could range from 7 to 28. A score of 26 was used as a cut-off because adherence was defined when the score was 27 or 28 (owing to deduction of 1 point from either of the ‘unintentional adherence’ questions — questions 1 or 6). Therefore, a score of 26 and below was considered to indicate non-adherence.^16^

### Operational definition

Uncontrolled hypertension was defined using an average of two office blood pressure readings according to the following cut-off points^9^:

a) 140/90 mmHg for patients with hypertension
b) 140/80 mmHg for patients with diabetes
c) 130/80 mmHg for patients with ischaemic heart disease/cerebrovascular disease and renal impairment
d) 150/90 mmHg for patients aged >80 years

Complementary alternative medication (CAM) was defined as herbal and dietary supplement consumption to control hypertension.

### Data analysis

All data obtained were analysed using the SPSS software version 21. We described the findings as frequencies, percentages, means, medians or standard deviations. Continuous data were described as means when data distribution was normal or medians when it was not. The chi-square test was used to determine the association between categorical data. An independent t-test or the Mann—Whitney test was used to determine the association between continuous and two categorical variables. Multivariate logistic regression (MLR) was performed to identify the determinants of medication non-adherence. The backward likelihood ratio model was selected over the forward method for the data analysis. This is because the forward method has a higher risk of type II errors due to suppressor effects; the likelihood ratio is less intense; and the Wald statistic can be unreliable in certain circumstances. In the backward likelihood ratio model, all variables were initially entered into the baseline model. Insignificant variables were then dropped, one at a time, until all the variables that remained in the model were statistically significant. This was conducted to identify the predictors of adherence in the final model after adjusting for confounders.

The assumptions for MLR were met in this study. First, the dependent variable was a dichotomous variable (yes/no for adherence). Second, there was no data duplication, while there was a separation of sample group outcomes. Third, there was no multicollinearity in the form of tolerance test (all independent variables, >0.1) and variance inflation factor (all independent variables, <5) detected among the independent variables. Fourth, the goodness of fit of the model was tested using the Hosmer and Lemeshow test. The P-value was 0.9, indicating that the model had a good fit. Last, our sample size (n=488) exceeded the calculated sample size of 457.

The independent variables with P-values of <0.25 in the univariate analyses were entered into the MLR to identify the determinants of the dependent variables. A P-value of <0.25 was adopted instead of a P-value of <0.05 to allow more significant variables to be included in the MLR. P-values of <0.05 were considered statistically significant.

## Results

A total of 501 patients with uncontrolled blood pressure were enrolled in the study. Thirteen patients were excluded from the analysis because of incomplete data. Thus, 488 patients were finally included in the data analysis. The prevalence of medication non-adherence was 39.3%. **Table 1** shows the socio-demographic characteristics of the study population. The mean patient age was 58.3±11.9 years. More than half of the patients were women (57.4%) and married (76.2%). The most common patient ethnicity was Chinese (39.1%), followed by Iban, Bidayuh, Melanau and Orang Ulu (35.5%), Malay (25.2%) and Indian (0.2%). Only 12.9% of the patients never had formal education. Approximately 42.4% were employed or had their own business. Approximately 82.4% had a monthly household income below RM 3,000 (USD 675), which is the median income for the bottom 40% of the income household group according to the Household Income and Basic Amenities Survey conducted in 2016 by the Department of Statistics.^19^

**Table 1** also shows the association between medication non-adherence and sociodemographic data among the respondents with uncontrolled hypertension in the univariate analysis. Younger patients and patients with a higher educational level were more likely to be non-adherent to antihypertensive medications. Meanwhile, retired or pensioners were more adherent to antihypertensive medications. However, no significant association was found between medication non-adherence and sex, ethnicity, monthly household income and marital status.

**Table 1:** Comparison of the adherence status towards antihypertensive medications in relation to the socio-demographic data among patients with uncontrolled blood pressure (n=488).

Variables	All	Adherence	Non-adherence	P-value
Age (year), meantSD	58.3±11-9	59-8±l 1.5	56.0±12.2	0.001
*Sex*				0.248
Male	208 (42.6)	120 (57.7)	88 (42.3)	
Female	280 (57.4)	176 (62.9)	104(37.1)	
*Ethnicity*				0.631
Malay	123 (25.2)	75 (61.0)	48 (39.0)	
Chinese	191 (39.1)	114(59-7)	77 (40.3)	
Indian	1 (0.2)	0 (0.0)	1 (100.0)	
Others	173 (35-5)	107 (61.8)	66 (38.2)	
*Educational level*				0.001
Primary school	151 (30.9)	87 (57.6)	64 (42.4)	
Secondary school	229 (46.9)	136 (59.4)	93 (40.6)	
College/university	45 (9.2)	21 (46.7)	24 (53.3)	
No formal education	63 (12.9)	52 (82.5)	11 (17.5)	
*Marital status*				0.887
Single	39 (8.0)	25 (64.1)	14 (35-9)	
Married	372 (76.2)	222 (59-7)	150 (40.3)	
Divorced	16 (3-3)	10 (62.5)	6 (37-5)	
Widowed	61 (12.5)	39 (63-9)	22 (36.1)	
*Employment status*				0.045
Employed/own business	207	115(55.6)	92 (44.4)	
Unemployed	183	112(61.2)	71 (38.8)	
Retired/pensioner	98	69 (70.4)	29 (29.6)	
*Monthly household income (MRY)*				0.823
<3,000 (USD <675)	402	246 (61.2)	156 (38.8)	
3,000-6,500 (USD 675-1,462)	68	41 (60.3)	27 (39-7)	
6,501-16,000 (USD 1,463-3,600)	14	7 (50.0)	7 (50.0)	
>16,000 (USD >3,600)	4	2 (50.0)	2 (50.0)	
*Time to a clinic visit (minute), median (IQR)*	15.0(10)	15.0(10)	15.0(10)	0.534

SD, standard deviation; IQR, interquartile range. Analysis was performed using the Mann— Whitney test, independent t-test or chi-square test.

**Table 2** shows the clinical characteristics of the study population. The mean systolic blood pressure was 151.1±11.0 mmHg, while the mean diastolic blood pressure was 87.4±9.9 mmHg. Approximately 71.5% of the patients had underlying dyslipidaemia. Only 33.4% had practiced home blood pressure monitoring. Calcium channel blockers were the most frequently prescribed antihypertensive agent (72.1%), followed by angiotensin-converting enzyme inhibitors (49.4%) and beta-blockers (37.1%). Approximately 10.9% of the patients had taken CAM. Table 2 also illustrates the association between medication non-adherence and clinical variables among the respondents with uncontrolled hypertension in the univariate analysis. Higher systolic and diastolic blood pressures and traditional CAM use were significantly associated with nonadherence. The patients who took angiotensin II receptor blockers (ARBs) and those with underlying ischaemic heart disease were more likely to adhere to antihypertensive medications.

**Table 2:** Comparison of the adherence status towards antihypertensive agents in relation to the clinical profiles among patients with uncontrolled blood pressure (n=488).

Variables	All	Adherence	Non-adherence	P-value
SBP (mmHg), mean±SD	151±11	150.3±10.9	152.3±11.2	0.047
DBP (mmHg), mean±SD	87±10	86.5±9.5	88.7±10.4	0.016
BMI (kg/m2), mean±SD	28.4±5.6	28.1±5.7	28.7±5.4	0.298
Duration of hypertension (year), median (IQR)	6.0 (10)	6.0 (11.0)	6.0 (8.0)	0.232
Antihypertensive agent, median (IQR)	2.0 (2.0)	2.0 (2.0)	2.0 (2.0)	0.994
*Type of antihypertensive medication (n, %)*				
Calcium channel blocker	352	221 (62.8)	131 (37.2)	0.122
ACEI	241	136 (56.4)	105 (43.6)	0.059
Beta-blocker	181	112 (61.9)	69 (38.1)	0.671
Diuretics	99	57 (57.6)	42 (42.4)	0.482
ARB	64	46 (71.9)	18 (28.1)	0.049
Alpha-blocker	9	6 (66.7)	3 (33.3)	0.709
*Co-morbidities (n, %)*				
Dyslipidaemia	349	216 (61.9)	133 (38.1)	0.376
Diabetes mellitus	184	104 (56.5)	80 (43.5)	0.146
Ischaemic heart disease	38	29 (76.3)	9 (23.7)	-0.04
Chronic kidney disease	31	16 (51.6)	15 (48.4)	0.287
Stroke	7	5 (71.4)	2 (28.6)	0.557
Home blood pressure monitoring (n, %)	163	99 (60.7)	64 (39.3)	0.979
Complementary alternative medication (n, %)	53	25 (47.2)	28 (52.8)	0.033
Satisfaction with clinic service, very poor to fair (n, %)	60 (12.3)	32 (53.3)	28 (46.7)	0.302
good to very good (n, %)	428 (87.7)	264 (61.7)	164 (38.3)	

SBP, systolic blood pressure; DBP, diastolic blood pressure; BMI, body mass index; ACEI, angiotensin-converting enzyme inhibitor; ARB, angiotensin II receptor blocker; SD, standard deviation; IQR, interquartile range. Analysis was performed using the Mann— Whitney test, independent t-test or chi-square test. Complementary alternative medication was defined as the consumption of herbal and dietary supplements to control hypertension.

**Table 3** shows the determinants of medication non-adherence in the MLR. The patients with tertiary educational level were more likely to be non-adherent than those without formal education (odds ratio [OR] =4.21, 95% CI=1.67-10.61, P=0.010).

The patients who consumed CAM were more likely to be non-adherent (OR=2.03, 95% CI=1.12–3.69, P=0.020) than those who did not. Non-usage of calcium channel blockers (OR=1.57, 95% CI= 1.02-2.41, P=0.039) and 1 mmHg increase in the systolic blood pressure were associated with higher odds of medication non-adherence (0R=1.03, 95% CI=1.00-1.05, P=0.006).

There was no significant association found between medication non-adherence and age, employment status, diastolic blood pressure, duration of hypertension, presence of diabetes mellitus, presence of ischaemic heart disease and usage of angiotensin-converting enzyme inhibitors or ARBs.

**Table 3:** Determinants of non-adherence towards antihypertensive agents among patients with uncontrolled blood pressure in the multivariate logistic regression (n=488).

Variables	Odds ratio	95% Cl		P-value
		Lower	Upper	
Age	0.973	0.955	0.99	0.003
*Education*				0.01
Primary school	3.095	1.472	6.505	0.003
Secondary school	2.52	1.206	5.266	0.014
College/university	4.213	1.672	10.614	0.002
No formal education	1			
*Employment*				0.539
Employed/own business (1)	1.295	0.689	2.435	0.421
Unemployed	1.422	0.765	2.646	0.266
Retired/pensioner	1			
Mean SBP	1.026	1.007	1.045	0.006
Mean DBP	1.006	0.983	1.029	0.624
Duration of hypertension	0.997	0.968	1.027	0.866
Use of ACEI, yes	1.022	0.658	1.586	0.924
*no*	1			
Use of calcium channel blocker, no	1.571	1.024	2.409	0.039
*yes*	1			
Use of ARB, no	1.593	0.863	2.94	0.137
*yes*	1			
Presence of diabetes mellitus, yes	1.378	0.924	2.054	0.116
*no*	1			
Presence of ischaemic heart disease, no	2.236	0.992	5.038	0.052
*yes*	1			
Use of complementary alternative medication, yes	2.029	1.115	3.69	0.02
*no*	1			
Clinic satisfaction, very poor to fair	1.152	0.64	2.073	0.637
*good to very good*	1			

SBP, systolic blood pressure; DBP, diastolic blood pressure; ACEI, angiotensin-converting enzyme inhibitor; ARB, angiotensin II receptor blocker; CI, confidence interval; Exp (B), exponentiation of the B coefficient. Complementary alternative medication was defined as the consumption of herbal and dietary supplements to control hypertension. *p<0.05

## Discussion

Herein, the prevalence of medication nonadherence was 39.3%. Surprisingly, this prevalence is low compared with previous reports. In a previous meta-analysis, a higher prevalence of non-adherence towards medications of 83.7% (95% CI=59.9-117.0) was noted in patients with uncontrolled hypertension.^20^ A high prevalence was also found in Portugal, whereby 51.8% were nonadherent to antihypertensive medications, and 67% had uncontrolled hypertension.^21^ Such differences between studies could be attributed to the differences in the settings and locations used.^21^

One-third of our study population was Chinese, and all of them adhered to the antihypertensive medications. We share similar results with Ramli et al., as their Chinese populations were also shown to have better adherence to antihypertensive medications.^16^ Thus, the prevalence in our study is lower. Three major factors affect uncontrolled hypertension: patient-, clinician- and healthcare system-related factors.^11,12^ In this study, medication non-adherence is mainly referred to patient-related factors.^11,12^

Nonetheless, clinician-related factors might have also affected the number of patients with uncontrolled hypertension in our study. This is supported by a local study in Negeri Sembilan, which found no relationship between adherence level and blood pressure control.^22^ The clinician’s failure to initiate or intensify therapy might have affected the results.^11,12,22^ This could also explain the lower non-adherence prevalence in our study. Further, only patients who visited the clinic for medication review and follow-up were recruited in this study.

Patients who defaulted on their follow-ups are likely to be non-compliant with medications and treatment advice.^23^ As defaulters were not included in our study, such selection bias might reduce the prevalence of non-adherence medication in patients with uncontrolled hypertension. According to the National Health and Morbidity Survey in 2019, 3 of 10 people in Malaysia have hypertension, and 50% remain undiagnosed. Approximately 90% of patients diagnosed with hypertension are on medication. However, only 45% have their blood pressure under control at <140/90 mmHg.^2^

Our study showed that patients with higher formal educational levels were more likely to be non-adherent with antihypertensive medications than those who did not receive any formal education. This finding is consistent with other studies.^6,24^ Boima et al. reported that patients who had received any form of education were more likely to show medication non-adherence than those who had not.^24^ Similarly, a Ghanaian study showed a significant negative association between educational status and medication.^6^ This may be explained by the fact that educated patients are more sceptical towards using antihypertensive medications.^6,24^

Herein, most patients on traditional CAM were not compliant with antihypertensive medications. Tan et al. showed similar findings that patients with hypertension who consumed CAM were less compliant than those who did not.^25^ Two other studies demonstrated similar findings. Both showed that those who took traditional medications were non-adherent to their antihypertensive medication regimen.^26,27^ The possible reasons were the belief that traditional medications must not be taken together with prescribed antihypertensives and lack of awareness about the danger of uncontrolled hypertension.^25^ The concern about western drug side effects and the belief that traditional CAM has fewer negative side effects may also contribute towards the higher level of non-adherence to antihypertensives among CAM users.^27^ This study also showed that higher systolic blood pressures were associated with non-adherence to antihypertensive medications. This is supported by the findings of Ramli et al., in which a poor adherence rate was found to negatively affect blood pressure control.^12^ Similarly, patients who were not adherent to antihypertensive agents were not put on calcium channel blockers.

Calcium channel blockers have been reported to be effective, safe and well tolerated in lowering blood pressure.^28^

### Strengths and limitations

The strength of this study was that it provides a better understanding of medication nonadherence among rural communities and primary care clinics in Sarawak, as data on this population are limited. However, the limitation of the study was its design. As this was a crosssectional study, only an association and not a causation could be inferred. There was also selection bias whereby defaulters were not recruited in this study, as they were found to be non-adherent. In addition, the blood pressure readings taken during the sampling day might not represent the usual or actual blood pressure of the participants; their blood pressure could be higher owing to insufficient rest, anxiety or concurrent infections.

## Conclusion

Medication non-adherence is present in nearly 40% of patients with uncontrolled blood pressure in Sarawak. Higher educational level, CAM usage and higher systolic blood pressure were associated with an increased risk of medication non-adherence. Hence, primary care physicians should be more vigilant in identifying patients at risk of non-adherence for further intervention of uncontrolled blood pressure.
